# Convection-Induced vs. Microwave Radiation-Induced in situ Drug Amorphization

**DOI:** 10.3390/molecules25051068

**Published:** 2020-02-27

**Authors:** Nele-Johanna Hempel, Matthias M. Knopp, Ragna Berthelsen, Korbinian Löbmann

**Affiliations:** 1 University of Copenhagen, Department of Pharmacy, 2100 Copenhagen, Denmarkragna.berthelsen@sund.ku.dk (R.B.); 2 Bioneer: FARMA, Department of Pharmacy, 2100 Copenhagen, Denmark

**Keywords:** in situ amorphization, microwave heating, convection heating, amorphous solid dispersion, glass solution

## Abstract

The aim of the study was to investigate the suitability of a convection oven to induce in situ amorphization. The study was conducted using microwave radiation-induced in situ amorphization as reference, as it has recently been shown to enable the preparation of a fully (100%) amorphous solid dispersion of celecoxib (CCX) in polyvinylpyrrolidone (PVP) after 10 min of continuous microwaving. For comparison, the experimental setup of the microwave-induced method was mimicked for the convection-induced method. Compacts containing crystalline CCX and PVP were prepared and either pre-conditioned at 75% relative humidity or kept dry to investigate the effect of sorbed water on the amorphization kinetics. Subsequently, the compacts were heated for 5, 10, 15, 20, or 30 min in the convection oven at 100 °C. The degree of amorphization of CCX in the compacts was subsequently quantified using transmission Raman spectroscopy. Using the convection oven, the maximum degree of amorphization achieved was 96.1% ± 2.1% (*n* = 3) for the conditioned compacts after 30 min of heating and 14.3% ± 1.4% (*n* = 3) for the dry compacts after 20 min of heating, respectively. Based on the results from the convection and the microwave oven, it was found that the sorbed water acts as a plasticizer in the conditioned compacts (i.e., increasing molecular mobility), which is advantageous for in situ amorphization in both methods. Since the underlying mechanism of heating between the convection oven and microwave oven differs, it was found that convection-induced in situ amorphization is inferior to microwave radiation-induced in situ amorphization in terms of amorphization kinetics with the present experimental setup.

## 1. Introduction

In situ drug amorphization is a novel approach to circumvent potential physical and chemical stability issues of amorphous solid dispersions (ASDs) during manufacturing and storage [1,2,3]. Using this approach, a crystalline drug is formulated together with a polymer into a final dosage form, for example, a compact, which subsequently can be “activated” into the amorphous form as a final manufacturing step or immediately prior to administration [2]. In situ amorphization of ASDs has been shown to be a promising concept, even though full amorphization was not achieved in several studies using either microwave radiation [2,4,5,6] or by immersion in water [7,8], with the model drugs celecoxib (CCX), indomethacin, ibuprofen, and naproxen. Only recently, 100% amorphization was achieved using the microwave oven [9].

In order to allow a successful amorphization of a compact using microwave radiation, a microwave absorbing material, for example, a dipolar molecule like water, is required to generate heat [10]. For this purpose, the compacts need to be conditioned at high relative humidity to allow sufficient water absorption, which in turn enables microwave heating. The heat is generated by friction caused by dipoles adapting to the electromagnetic field [10]. In contrast to convection-induced heating, microwave heating is also referred to as internal heating, as the energy of the microwave radiation is converted to heat uniformly (homogeneously) throughout the material. Here, only a small temperature gradient exists from the core of the material to the surface, due to a cooling effect of the surrounding air [10]. It has been suggested that the in situ amorphization process is driven by dissolution/fusion of the drug into the polymer at temperatures above the glass transition temperature (*T*_g_) of the polymer [9,11,12]. Since internal heating within the compact during microwaving induces the in situ amorphization process, it is fundamentally interesting whether heating using a convection oven would yield similar results. Compared to microwave heating, the heating mechanism of a heating chamber, such as a convection oven, is different as an external heating source convects the heat from outside towards the compacts, where the heat is then conducted to the core of the compact, which usually creates a greater temperature gradient than observed in the microwave oven [13]. The heat conduction is dependent on the thermal conductivity properties of the material. Hence, a convection oven is slower and less efficient than a microwave oven [10]. However, in a convection oven, heating can be achieved without the presence of a microwave absorbing material, i.e., using the convection oven as a heat source could potentially allow omitting the high humidity-conditioning step. On the other hand, convection-induced heating can be considered more inefficient, as it consumes large amounts of energy to heat a small compact [14].

In this study, the suitability of the convection-induced in situ amorphization approach was investigated. To allow a direct comparison, the microwave oven setup described by Hempel et al. (2020) was mimicked with respect to the maximum temperature reached during heating (100°C) [9]. Furthermore, the influence of sorbed water was studied both as a source for heat conductivity and as a plasticizer. In short, CCX and PVP were subjected to ball milling and sieving to obtain small particle sizes (< 71 µm). Subsequently, physical mixtures of 30% (*w/w*) CCX, 69.5% (*w/w*) PVP, and 0.5% (*w/w*) magnesium stearate (lubricant) were pressed into compacts of approximately 100 mg. The compacts were either stored at 75% relative humidity for two weeks (conditioned compacts) or used immediately (dry compacts). The dry and conditioned compacts were placed in a convection oven at 100 °C for different times, whilst the temperature of the core of the compacts was monitored. Before and after exposure, the degree of amorphization was quantified using transmission Raman spectroscopy.

## 2. Results and Discussions

The obtained particle sizes after sieving for CCX and PVP were *d*_0.5_ = 6.6 µm and *d*_0.5_ = 5.0 µm, respectively. Prior to heating, the conditioned compacts contained ~22% (*w/w*) of water determined by weight gain, whereas the dry compacts only contained the bulk water content of the PVP corresponding to ~4% (*w/w*) in the compact. Due to the high water content in the conditioned compacts, the compacts became soft. As sorbed water in the compacts has previously shown to act as a plasticizer through decreasing the *T*_g_ of the polymer and increasing molecular mobility [2], the dissolution rate of the drug into the polymer during heating was proposed to increase with increasing water content [2,9].

**Figure 1:**
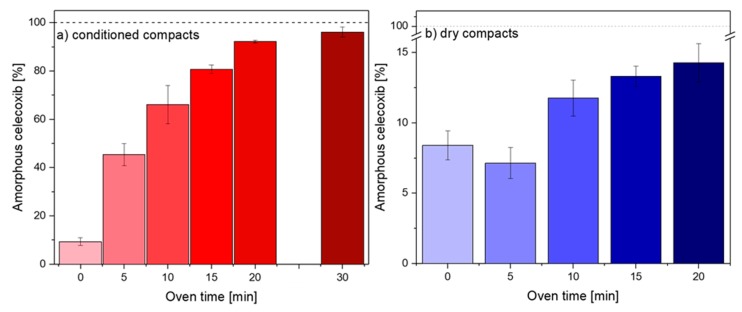
Degree of amorphization of celecoxib (CCX) quantified by transmission Raman spectroscopy for (**a**) conditioned (red) and (**b**) dry (blue) compacts after different times in the convection oven (0, 5, 10, 15, 20 and 30 (only Figure 1a) min). Data represents mean ± SD (*n* = 3, *n* = 12 (0 min)).

Figure 1 shows the degree of amorphization achieved for the conditioned and dry compacts following convection heating for 0–30 min. As can be seen with increasing exposure time, the overall degree of amorphization increases, however, to a different extent for the conditioned and dry compacts, respectively. When subjected to 20 min of heating in the convection oven at 100 °C, the conditioned compacts showed a degree of amorphization of 92.2% ± 0.2% (*n* = 3), whilst the dry compacts reached 14.3% ± 1.4% (*n* = 3). Both sets of compacts showed small amounts of amorphous CCX prior to heating (0 min), i.e., 9.3% ± 1.6% (conditioned) and 8.4% ± 1.0% (dry) (*n* = 12). The small amount of initial amorphization may be due to the applied compaction pressure and/or occur during conditioning, as similar results have previously been reported [1,9,15].

In an attempt to calculate the necessary exposure time to obtain complete amorphization of the conditioned compacts through convection, a mathematical data fit was conducted using the data displayed in Figure 1a (0–20 min). This was not attempted for dry compacts, as the required time to reach full amorphization was expected to be unpractical and is even suggested to be not possible at 100 °C (Figure 1b). Based on the mathematical fit, the degree of amorphization as a function of exposure time was found to follow a first-order kinetic:

(1) $$y=a-b\times c^{x}$$

This observation correlates well with the proposed mechanism of amorphization, i.e., the rate limiting step is assumed to be the drug dissolution process into the softened polymer [9], which is also a first-order process, as described by Noyes and Whitney in 1897 [16]. Upon extrapolation of the fitted function to 100% amorphization (Figure S1), a complete ASD should theoretically be obtained after 25.3 min in the convection oven (Figure S1). Note that this assumption does not take into account that the properties of the compact will change during heating, for example, water content due to evaporation, and as a result hereof, the *T*_g_ of the system. Hence, to allow sufficient time to obtain full amorphization, the conditioned compacts were heated in the convection oven for 30 min. The degree of amorphization was found to be slightly higher than after 20 min with 96.1 ± 2.1% (*n* = 3) after 30 min, but a full amorphization was not achieved (Figure 1a).

Using PLM imaging, it was found that the remaining crystallinity was mostly at the surface of the compact, where most water evaporation is expected to take place. Only some small crystals remained in the core of the compacts after 30 min in the convection oven (Figure S2). As water evaporation increases the *T*_g_ of the polymer and decreases its mobility, it is assumed that the crystalline drug was unable to dissolve into the “dried” polymer. These findings are supported by Edinger et al. (2018), who reported residual crystallinity on the surface of compacts following microwave radiation-induced in situ amorphization [4].

**Figure 2:**
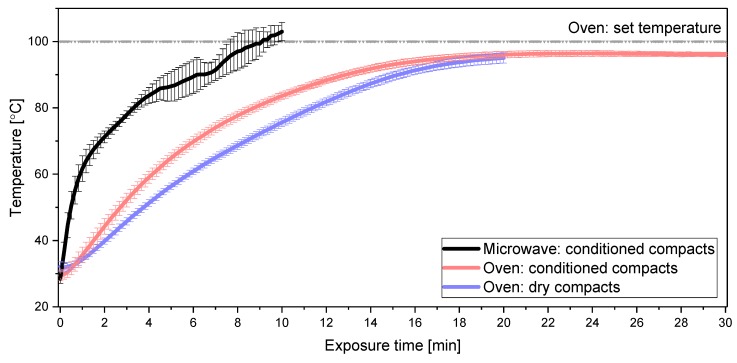
Comparison of the temperature profiles obtained during microwaving of conditioned compacts (black) (see Hempel et al. (2020) [9]) and during convection heating for conditioned (red) and dry (blue) compacts; dotted line (grey) indicates the set oven temperature; temperature (°C) plotted against the exposure time (min); data represents mean ± SD (*n* = 3).

Dry compacts (Figure 1b) showed overall negligible amorphization following heating, i.e., even after 20 min in the convection oven, less than 15% of the CCX was amorphous. The observed decrease in the degree of amorphization from 0 to 5 min was statistically not significant (*p* > 0.05). The effect of the sorbed water on the amorphization process is two-fold: on the one hand, it acts as a plasticizer, lowering the *T*_g_ of the polymer, and on the other hand, it has a greater thermal conductivity than PVP due to stronger intramolecular interactions and structural order [17,18,19]. Hence, the temperature in the core of the conditioned compacts was expected to increase faster compared to the dry compacts. In accordance with this, the temperature profiles of the different compacts revealed a statistically significant difference (*p* < 0.05) between the temperatures from 3–16 min of heating obtained for conditioned and dry compacts (Figure 2).

The sorbed water in the conditioned compacts had a positive impact on the overall thermal conductivity of the compact. During convection heating, the heat is convected from the outside (surface) towards the core of the compact, resulting in a higher surface temperature [13]. The heat from the surface is transported into the core of the compact by conduction, which is dependent on the thermal conductivity of the material, for example, PVP and water. The core temperature reached between 95 and 96 °C for both sets of compacts after 20 or 30 min of heating, respectively, which is only slightly below the set temperature/surface temperature of the compact of 100 °C. For the conditioned compacts, the core temperature during heating was constantly above the *T*_g_ of the conditioned polymer (approximately −16 °C after conditioning) and the *T*_g_ of the resulting ASD (approximately 58 °C) generated during the in situ amorphization [9]. For the dry compacts, the temperature reached the *T*_g_ of the “dry” polymer (approximately 76 °C with ~4% *w/w* bulk water) after ~ 10 min, however, exceeded this temperature merely by a few degrees (< 20 °C after 20 min). Compared with the microwave oven, the increase in core temperature of the compacts in the convection oven experiments was much slower and did not reach the same temperature after 10 min despite a similar set temperature of the convection oven (Figure 2). Following 10 min of exposure in the convection oven, the temperature measured in the conditioned and dry compacts was ~20 °C and ~25 °C, respectively, below the measured temperature in conditioned compacts heated by a microwave oven. As the molecular mobility of the polymer increases with increasing temperatures above the *T*_g_, the polymer is more constrained at 10 min during convection oven heating compared to microwave heating. Hence, the dissolution/fusion process of CCX into the polymer is slower using the convection oven, making convection-induced heating less feasible for the in situ amorphization compared to the microwave oven using this experimental setup. It was also observed that the conditioned compacts heated by a microwave oven contain less water after 10 min (~5% *w/w* water) compared to the conditioned compacts heated by a convection oven after 20 min (~6.5% *w/w* water). Hence, the dehydration appears slower in a convection oven. Since water is favorable in both setups, this should, in theory, be advantageous for the convection oven. However, the results in this study suggest that the kinetics of the dehydration are playing a minor role compared to the temperature profiles obtained by either convection-induced or microwave-induced in situ amorphization.

## 3. Materials and Methods

### 3.1. Materials

Celecoxib (CCX, *M*_w_ = 381.37 g/mol) and magnesium stearate were purchased from Fagron Nordic A/S (Copenhagen, Denmark). Kollidon K12^®^ (PVP, *M*_w_ = 2000–3000 g/mol) was kindly supplied by BASF (Ludwigshafen, Germany). Sodium chloride (NaCl, *M*_w_= 58.44 g/mol) was purchased from Sigma-Aldrich (St. Louis, MO, USA). All chemicals were used as received.

### 3.2. Compact Preparation and Storage

CCX and PVP were separately subjected to milling for 1 min each. An oscillatory ball mill Mixer Mill MM400 from Retsch GmbH and Co. (Haan, Germany) was used at a frequency of 30 Hz using 25 mL jars containing two stainless steel balls with a diameter of 12 mm each at 4 °C. The milled CCX was subsequently stored at 40 °C overnight to ensure full crystallinity in a UF55 universal oven at 100 °C from Memmert (Schwabach, Germany). For particle size separation, CCX and PVP were separately sieved using a sieving tower from Fritsch GmbH (Ida-Oberstein, Germany) at an amplitude of 2 in the permanent mode using a 71 µm sieve for 5 min intervals until the weight of the sieve did not change by more than 5% in between two consecutive sieving intervals. Only the fraction <71 µm was used for this study. The particle size was determined using laser diffraction analysis. The analysis was performed on a Malvern Mastersizer 2000 with a Scirocco 2000 adapter for dry powder analysis from Malvern Panalytical Ltd. (Malvern, UK). The experiments were conducted under a pressure of 3 bar using approximately 1 g of each compound. Results are reported as the particle size *d*_0.5_ (µm) with the greatest volume (%). Subsequently, a physical mixture containing 30% (*w/w*) CCX, 69.5% (*w/w*) PVP, and 0.5% (*w/w*) magnesium stearate was obtained by gentle shaking in a glass vial. Using approximately 100 mg of the physical mixtures, flat-faced cylindrical compacts with a diameter of 6 mm were prepared at a compaction pressure of 35 MPa using an instrumented single punch tablet press GTP-1 from Gamlen Instruments (Nottingham, UK) fitted with a 500 kg load cell (CT6–500-022).

Half of the compacts were conditioned for 2 weeks at 75% relative humidity (saturated NaCl solution) at ambient temperature (conditioned), and the other half of the compacts were processed immediately (dry).

### 3.3. In situ Amorphization

In order to induce in situ amorphization, the compacts (dry or conditioned) were placed in a UF55 convection oven at 100 °C from Memmert (Schwabach, Germany). The compacts (dry or conditioned) were placed on polypropylene watch glass, and placed in the oven alongside 3 beakers containing a total of 1.3 L of distilled water with glass beads in Quick Clean™ microwave steam bags from Medela AG (Baar, Switzerland). The amount of water in the oven corresponds to the amount of water (500 mL) used in the microwave oven relative to its volume [9]. An additional beaker was placed upside down over the compact holders to protect the compact from increasing humidity inside the oven.

The compacts were subjected to convective heating for 5, 10, 15, 20 min, and additionally 30 min for conditioned compacts. During the heating for 20 (dry) and 30 min (conditioned), the temperature of the compacts was measured at the core of the compact every second with a temperature resolution of 0.1 °C. For the dry compacts, a small hole was drilled to the core to insert the fiber optic temperature probe OTG-A from Opsens Solutions (Québec, Canada). For the conditioned compacts, the probe was gently inserted into the core of the softened compact. The signal was conducted using a Pico-M signal conductor from Opsens Solutions (Québec, Canada) and analyzed using the Softsense Software from Opsens Solutions (Québec, Canada). Each experiment was conducted in triplicate.

### 3.4. Quantification of the Degree of Amorphization

In order to quantify the degree of amorphization of the compacts before and after heating, transmission Raman spectroscopy was used. A Kaiser RXN1 Microprobe from Kaiser Optical Systems (Ann Arbor, MI, USA) equipped with a PhaT-probe in a transmission Raman configuration setup was used as described previously by Edinger et al. (2018) [4]. A Thorlabs AD127NT adaptor (Newton, NJ, US) was situated on the excitation fiber. The compacts were directly placed on the adaptor. The inelastically scattered light was collected using a 5x objective with a PhaT-probe at a distance of 20 mm to the diffuser. Each spectrum had a total acquisition time of 20 s, which was an average of 5 measurements with an exposure time of 4 s each. The dark frames were subtracted for each measurement. At the fiber output, the wavelength was 785 nm with an excitation power of 200 mW. The Raman shift was measured from 150 to 1900 cm^−1^ at a resolution of 5 cm^−1^. The analysis was conducted using a calibration space and a partial least-squares regression (PLS) model, which is presented in and was kindly supplied by Edinger et al. (2018) [4]. In short, in the spectral region from 705 to 845 cm^−1^ distinguishing features are present to differentiate between the crystalline state and the amorphous form of CCX. A calibration space for the PLS model was obtained from 17 different mixtures containing crystalline CCX, amorphous CCX, and PVP. Preprocessing of the data was performed by Savitzky–Golay smoothing and standard normal variate transformation; all data processing was performed in MatLab from Mathworks (Natick, MA, USA) using the PLS toolbox 8.1.1 from Eigenvector Research Inc. (Manson, WA, USA). Cross-validation of the calibration space was performed.

### 3.5. Residual Crystallinity

The heated compacts were investigated for remaining crystallinity by carefully slicing the compacts with a scalpel into edge and core areas. A DM LM microscope from Leica Microsystems GmbH (Wetzlar, Germany) was used and operated in polarized light microscopy (PLM) mode using a 10× magnifying objective. The images were taken using an Evolution MP camera from Media Cybernetics (Rockville, MD, USA) controlled by the Image-Pro Insight software version 8.0.21 from Media Cybernetics (Rockville, MD, USA).

## 4. Conclusions

Overall, the convection oven displayed different heating profiles in the core of the compact compared to the microwave oven, most likely due to the difference in the underlying heating mechanism. Consequently, heat transfer through convection is suggested to be unfavorable for in situ amorphization, as it leads to residual surface crystallinity, even for conditioned compacts with a *T*_g_ below the set temperature. It is suggested that, due to a slower increase in temperature during the convection-induced heating, a drying process prevents a full amorphization, predominantly at the compact surface.

Hence, a convection oven is less feasible than a microwave oven for in situ amorphization when applying similar experimental conditions. Compared with microwave-induced in situ amorphization, no full ASD was obtained even after 30 min of heating at 100 °C.

## Supplementary Materials

The following are available online at https://www.mdpi.com/1420-3049/25/5/1068/s1, Figure S1: First-order kinetic fit plotting, Figure S2: PLM Images.
