# Supplementary material for: Convection-Induced vs. Microwave Radiation-Induced in situ Drug Amorphization
Source: Molecules. 2020 Feb 27;25(5):1068. doi: 10.3390/molecules25051068 (PMC7179224; doi:10.3390/molecules25051068)
Supplement: Supplementary file 1 [file molecules-25-01068-s001.pdf]

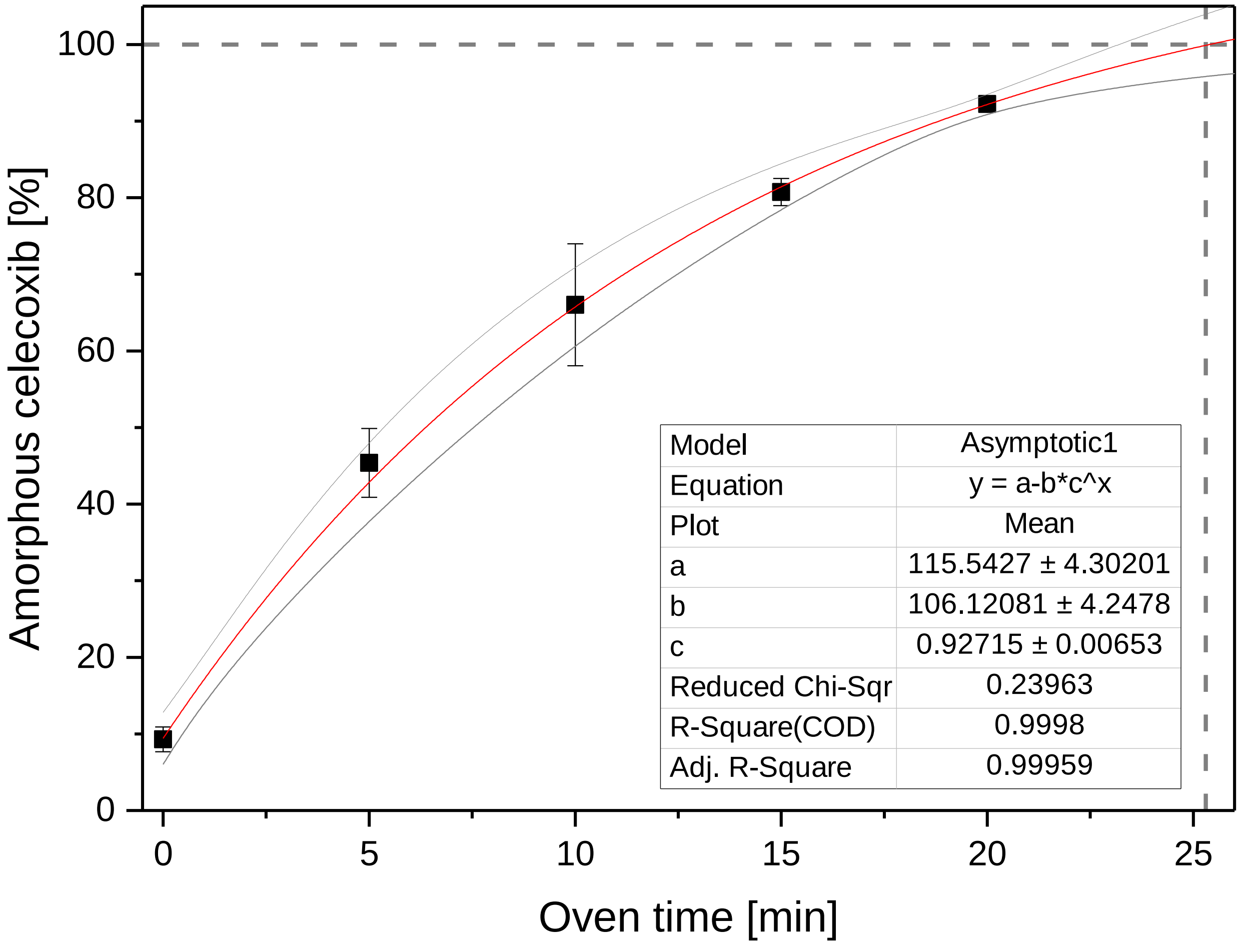

Figure S1. First-order-kinetic fit plotting the amorphous celecoxib content [%] reached in conditioned compacts against the oven time [min]. Solid grey lines indicate the confidence interval (95%). Dotted grey lines indicate the expected heating time to obtain a complete ASD.

$$y = (115.5427 \pm 4.30201) - (106.1208 \pm 4.24789) * ((0.92715 \pm 0.00653)^x); (Eq. 1);$$

$$x = time\ [min]; y = amorphization\ degree\ [\%]; R^2 = 0.99959$$

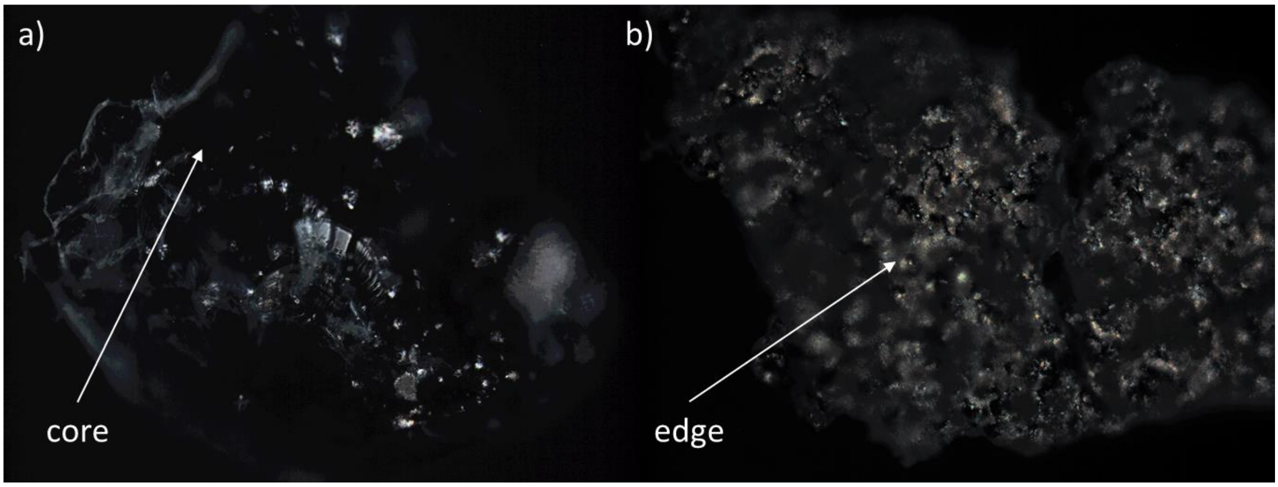

Figure S2. PLM images of heated conditioned compacts after 30 min; a) core area; b) edge area. The light structures indicate crystallinity.
